# Effects of *FSHR* polymorphisms on premature ovarian insufficiency in human beings: a meta-analysis

**DOI:** 10.1186/s12958-019-0528-1

**Published:** 2019-10-19

**Authors:** Wenling Huang, Ying Cao, Lei Shi

**Affiliations:** 1 Reproductive Endocrinology Center, Dongfang Hospital, Beijing University of Chinese Medicine, No. 6 Fangxingyuan 1st Block, Fengtai District, Beijing, China; 2 College of Traditional Chinese Medicine, North China University of Science and Technology, Hebei, China; 3 Research Office, Dongfang Hospital, Beijing University of Chinese Medicine, Beijing, China

**Keywords:** Premature ovarian insufficiency (POI), Follicle-stimulating hormone receptor (*FSHR*), Gene polymorphisms, Meta-analysis, Human beings, Asians

## Abstract

**Background:**

Whether follicle-stimulating hormone receptor (*FSHR*) polymorphisms are implicated in premature ovarian insufficiency (POI) remains controversial. Thus, we performed this study to explore correlation between *FSHR* polymorphisms and POI in human beings.

**Methods:**

Literature retrieve was conducted in PubMed, Medline, Embase and CNKI. Odds ratios (ORs) and 95% confidence intervals (CIs) were calculated.

**Results:**

Sixteen studies were enrolled for analyses. No significant relationship with POI was found for rs6165 and rs6166 polymorphisms in overall analyses. Further subgroup analyses revealed that rs6166 polymorphism was significantly associated with the risk of POI in Asians with both FEM and REM. Nevertheless, we failed to detect any significant associations with POI for other ethnicities.

**Conclusions:**

Our findings indicated that *FSHR* rs6166 polymorphism may serve as a potential genetic biomarker of POI in Asians, but not in other ethnicities.

## Background

Premature ovarian insufficiency (POI) is currently defined as apparent deterioration of ovarian function before the age of 40 in human beings [1]. It is characterized by an elevated level of follicle-stimulating hormone (FSH), a decreased level of estrogen, oligomenorrhea or amenorrhoea as well as an increased risk of osteoporosis and multiple cardiovascular diseases [2]. According to a recent epidemiological study, the prevalence of POI is estimated to be around 1% in women younger than 40 years old [3]. To date, the exact pathogenic mechanism of POI is still largely unknown. Nevertheless, there is mounting evidence to support that genetic factors play vital roles in its occurrence and development. First, family aggregation of POI in human beings is not uncommon, and it is estimated that about 10–30% of POI patients have positive family history [4]. Second, various genetic variants have already been found to be correlated with an increased risk of POI in human beings by previous experimental studies [5, 6]. In summary, these findings jointly indicated that genetic predisposition to POI is crucial for its development.

FSH is a glycoprotein secreted by the pituitary gland, and it plays a crucial role in promoting follicle growth and regulating ovarian function by acting on the FSH receptor (FSHR) [7]. Therefore, it is biologically plausible that functional *FSHR* polymorphisms may result in dysfunction of FSH, lead to decreased ovarian function and give rise to the development of POI in human beings. The rs6165 and rs6166 polymorphisms are two commonly seen missense mutations of *FSHR*, the G to A transvertion at these two loci lead to amino acid residue substitution of the corresponding amino acid sequence, and thus these two polymorphisms may affect bonding of FSH and FSHR [8]. Previous studies have shown that rs6165 and rs6166 polymorphisms were actually correlated with a higher serum FSH level and reduced FSH efficiency in human beings [9, 10]. Considering the functional significances of rs6165 and rs6166 polymorphisms, several pilot studies have already been conducted to investigate the possible correlation between these two polymorphisms and POI. However, the results of these studies were inconsistent and the sample size of individual studies was relatively small. Therefore, we conducted this meta-analysis to better analyze the roles of *FSHR* polymorphisms in POI.

## Methods

### Literature search and inclusion criteria

This meta-analysis was adhered to the Preferred Reporting Items for Systematic Reviews and Meta-analyses (PRISMA) guideline [11]. Potentially related literatures (published before September 2018) were retrieved from PubMed, Medline, Embase and China National Knowledge Infrastructure (CNKI) using the following searching strategy: (premature ovarian insufficiency OR premature ovarian failure OR POI OR POF) AND (polymorphism OR variant OR mutation OR genotype OR allele) AND (follicle stimulating hormone receptor OR FSHR). Furthermore, the references of retrieved articles were also screened for identification of other potentially relevant studies.

To test the research hypothesis of this meta-analysis, included studies must meet all the following criteria: a. case-control study on correlation between *FSHR* polymorphisms and POI in human beings; b. provide genotypic and/or allelic frequency of investigated *FSHR* polymorphisms; c. full text in English or Chinese available. Studies were excluded if one of the following criteria was fulfilled: a. not relevant to *FSHR* polymorphisms and POI in human beings; b. case reports or case series; c. abstracts, reviews, comments, letters and conference presentations. For duplicate publications, we only included the study with the largest sample size for analyses.

### Data extraction and quality assessment

The following data were extracted from all included studies: (1) name of first author; (2) year of publication; (3) country and ethnicity of participants; (4) the number of cases and controls; and (5) the genotypic distribution of *FSHR* polymorphisms in cases and controls. Additionally, the probability value (*P* value) of Hardy-Weinberg equilibrium (HWE) test was also calculated.

The Newcastle-Ottawa scale (NOS) was employed to assess the quality of eligible studies from three aspects: (1) selection of cases and controls; (2) comparability between cases and controls; and (3) exposure in cases and controls [12]. The NOS has a score range of zero to nine, and studies with a score of more than seven were thought to be of high quality.

Two reviewers conducted data extraction and quality assessment independently. When necessary, the reviewers wrote to the corresponding authors for extra information or raw data. Any disagreement between two reviewers was solved by discussion until a consensus was reached.

### Statistical analysis

All statistical analyses in the present study were conducted with Review Manager Version 5.3.3 (The Cochrane Collaboration, Software Update, Oxford, United Kingdom). ORs and 95% CIs were used to assess the strength of correlation between *FSHR* polymorphisms and POI in all possible genetic models, and a *p* value of 0.05 or less was considered to be statistically significant. Between-study heterogeneities were evaluated by Q test and I^2^ statistic. If *p* value of Q test was less than 0.1 or I^2^ was greater than 50%, between-study heterogeneities were considered to be obvious. Subgroup analyses by ethnicity of participants were subsequently conducted to obtain more specific results. Overall and subgroup analyses were performed with both fixed-effect models (FEMs) and random-effect models (REMs). Sensitivity analyses were carried out to test the stability of the results. Funnel plots were applied to evaluate possible publication bias.

## Results

### Characteristics of included studies

**Fig. 1:**
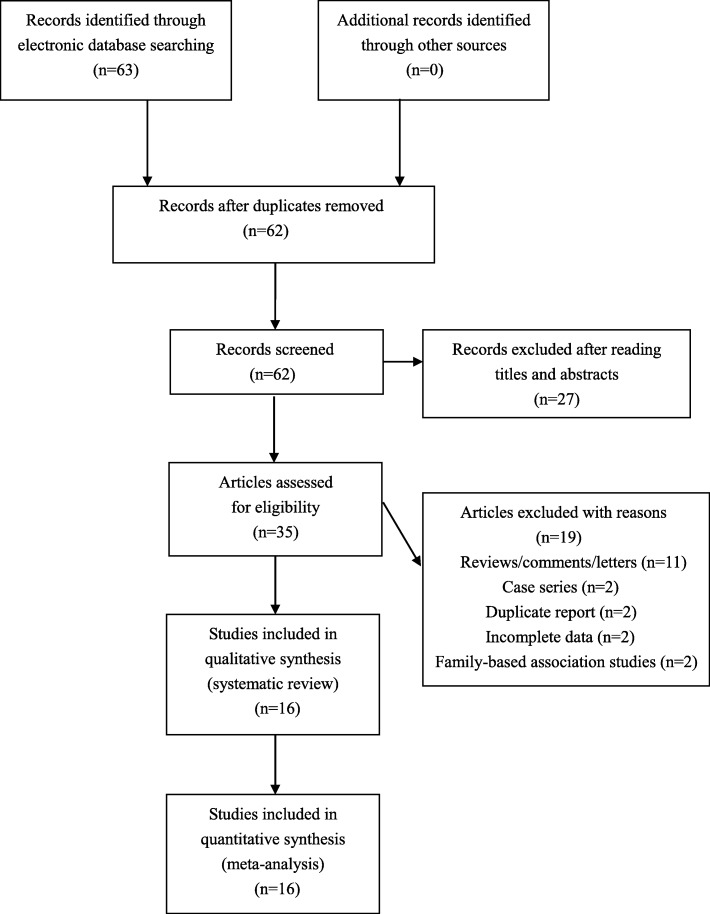
Flowchart of study selection for the present study

The literature search identified 63 potentially relevant articles. After exclusion of irrelevant and duplicate articles by reading titles and abstracts, 35 articles were retrieved for further evaluation. Another 19 articles were subsequently excluded after reading the full text. Finally, a total of 16 studies that met the inclusion criteria of our meta-analysis were included (see Fig. 1). Characteristics of included studies were summarized in Table 1.

**Table 1:** The characteristics of included studies

First author, year	Country	Ethnicity	Age (years) Case/Control	Sample size Case/Control	Genotype distribution		*P*-value for HWE	NOS score
					Cases	Controls		
rs6165 A/G								
Bretherick 2008	Canada	Mixed	35.5/35.0	55/105	NA	NA	NA	7
Conway 1999	UK	Caucasian	NA/NA	49/51	NA	NA	NA	7
Cordts 2015	Brazil	Mixed	31.0/31.0	96/123	29/48/19	55/53/15	0.687	7
da Fonte Kohek 1998	Brazil	Mixed	22.2/31.0	15/42	3/8/4	13/24/5	0.228	7
Du 2010	China	Asian	NA/NA	40/92	20/18/2	40/37/15	0.207	7
Ghezelayagh 2018	Iran	Mixed	NA/NA	84/80	24/50/10	28/48/4	0.004	8
Juárez-Rendón 2018	Mexico	Mixed	30.0/27.0	20/50	8/10/2	26/17/7	0.147	8
Liu 1998	Germany	Caucasian	NA/NA	5/4	2/3/0	3/1/0	0.775	7
Ma 2015	China	Asian	29.8/29.3	63/58	33/25/5	28/25/5	0.861	7
Sudo 2002	Japan	Asian	32.9/31.3	17/163	NA	NA	NA	7
Sundblad 2004	Argentina	Caucasian	27.0/27.2	15/44	NA	NA	NA	7
Tong 2001	Singapore	Asian	24.9/25.4	16/236	2/13/1	102/110/24	0.475	8
Vilodre 2008	Brazil	Mixed	NA/NA	35/42	9/19/7	13/24/5	0.228	7
Woad 2013	New Zealand	Caucasian	NA/NA	80/80	28/34/18	25/38/17	0.718	7
rs6166 A/G								
Bretherick 2008	Canada	Mixed	35.5/35.0	55/105	NA	NA	NA	7
Conway 1999	UK	Caucasian	NA/NA	49/51	NA	NA	NA	7
Cordts 2015	Brazil	Mixed	31.0/31.0	96/123	30/52/14	34/68/21	0.190	7
da Fonte Kohek 1998	Brazil	Mixed	22.2/31.0	15/42	5/7/3	13/24/5	0.228	7
Du 2010	China	Asian	NA/NA	37/90	16/19/2	40/34/16	0.077	7
Kim 2011	South Korea	Asian	NA/NA	83/176	23/51/9	67/89/20	0.238	7
Ma 2015	China	Asian	29.8/29.3	63/58	31/30/2	32/22/4	0.934	7
Sudo 2002	Japan	Asian	32.9/31.3	17/168	NA	NA	NA	7
Sundblad 2004	Argentina	Caucasian	27.0/27.2	15/44	NA	NA	NA	7
Tong 2001	Singapore	Asian	24.9/25.4	16/236	5/11/0	91/132/13	< 0.001	8
Vilodre 2008	Brazil	Mixed	NA/NA	35/42	15/15/5	13/24/5	0.228	7
Woad 2013	New Zealand	Caucasian	NA/NA	80/80	29/33/18	26/37/17	0.572	7
Yin 2016	China	Asian	31.3/29.2	79/118	25/37/17	43/45/30	0.013	8

Abbreviations: *HWE* Hardy-Weinberg equilibrium, *NOS* Newcastle-Ottawa scale, *NA* Not available

### Overall and subgroup analyses

To investigate potential correlations between *FSHR* polymorphisms and POI in human beings, 14 studies about rs6165 polymorphism (590 cases and 1170 controls) and 13 studies about rs6166 polymorphism (640 cases and 1333 controls) were enrolled for analyses. No significant relationship with POI was found for two investigated polymorphisms in overall analyses. Further subgroup analyses by ethnicity revealed that rs6166 polymorphism was significantly associated with the risk of POI in Asians with both FEM (additive model: *p* = 0.005, OR = 1.55, 95% CI 1.14–2.09) and REM (additive model: *p* = 0.005, OR = 1.55, 95% CI 1.14–2.09). Nevertheless, we failed to detect any significant associations with POI for other ethnicities (see Table 2).

**Table 2:** Results of overall and subgroup analyses for *FSHR* gene polymorphisms and POI

Population	Sample size Case/Control	Dominant comparison			Recessive comparison			Additive comparison			Allele comparison		
		*P* value	OR (95% CI)	I^2^ statistic	*P* value	OR (95% CI)	I^2^ statistic	*P* value	OR (95% CI)	I^2^ statistic	*P* value	OR (95% CI)	I^2^ statistic
rs6165 A/G													
Overall (FEM)	590/1170	0.05	0.77 (0.60–1.00)	18%	0.26	1.23 (0.86–1.76)	11%	0.25	1.16 (0.90–1.48)	7%	0.07	0.87 (0.74–1.01)	3%
Overall (REM)	590/1170	0.12	0.79 (0.58–1.06)	18%	0.24	1.28 (0.85–1.93)	11%	0.34	1.14 (0.87–1.48)	7%	0.07	0.86 (0.74–1.01)	3%
Asian (FEM)	136/549	0.72	0.92 (0.58–1.46)	64%	0.13	0.52 (0.22–1.22)	0%	0.20	1.36 (0.85–2.16)	63%	0.86	0.97 (0.71–1.33)	51%
Asian (REM)	136/549	0.68	0.83 (0.34–2.01)	64%	0.20	0.56 (0.23–1.35)	0%	0.35	1.50 (0.64–3.48)	63%	0.72	0.92 (0.58–1.45)	51%
Caucasian (FEM)	149/179	0.81	1.08 (0.57–2.04)	19%	0.85	1.08 (0.51–2.28)	NA	0.71	0.89 (0.49–1.63)	22%	0.89	0.98 (0.71–1.34)	0%
Caucasian (REM)	149/179	0.92	0.95 (0.31–2.89)	19%	0.85	1.08 (0.51–2.28)	NA	0.94	1.05 (0.32–3.41)	22%	0.90	0.98 (0.71–1.35)	0%
rs6166 A/G													
Overall (FEM)	640/1333	0.52	0.92 (0.73–1.17)	0%	0.29	0.84 (0.61–1.16)	0%	0.16	1.18 (0.94–1.48)	9%	0.87	1.01 (0.88–1.17)	0%
Overall (REM)	640/1333	0.54	0.93 (0.73–1.18)	0%	0.39	0.86 (0.62–1.20)	0%	0.19	1.18 (0.92–1.50)	9%	0.89	1.01 (0.87–1.17)	0%
Asian (FEM)	295/846	0.08	0.76 (0.55–1.03)	0%	0.12	0.69 (0.43–1.10)	0%	**0.005**	**1.55 (1.14–2.09)**	0%	0.46	0.93 (0.75–1.14)	0%
Asian (REM)	295/846	0.68	0.83 (0.34–2.01) 64%		0.20	0.56 (0.23–1.35)	0%	**0.005**	**1.55 (1.14–2.09)**	0%	0.45	0.92 (0.75–1.14)	0%
Caucasian (FEM)	144/175	0.62	1.18 (0.61–2.27)	NA	0.85	1.08 (0.51–2.28)	NA	0.52	0.82 (0.44–1.53)	NA	0.99	1.00 (0.72–1.37)	0%
Caucasian (REM)	144/175	0.62	1.18 (0.61–2.27)	NA	0.85	1.08 (0.51–2.28)	NA	0.52	0.82 (0.44–1.53)	NA	0.99	1.00 (0.72–1.37)	0%

The values in bold represent there is statistically significant differences between cases and controls

Abbreviations: *OR* Odds ratio, *CI* Confidence interval, *NA* Not available

### Sensitivity analyses

Sensitivity analyses were carried out to examine the stability of pooled results by eliminating studies that deviated from HWE. No changes of results were observed in any comparisons, which indicated that our findings were statistically reliable.

### Publication biases

Potential publication biases in the current study were evaluated with funnel plots. No obvious asymmetry of funnel plots was observed in any comparisons, which suggested that our findings were unlikely to be influenced by severe publication bias.

## Discussion

To the best of our knowledge, this is so far the most comprehensive meta-analysis on correlations between *FSHR* polymorphisms and POI. The overall and subgroup analyses revealed that the rs6166 polymorphism was significantly associated with the risk of POI in Asians under additive comparison. But we failed to detect any positive results for other ethnicities. The stability of the synthetic results was subsequently evaluated in sensitivity analyses, and no changes of results were observed in any comparisons, which indicated that our findings were quite stable and reliable.

There are several points that need to be addressed about this meta-analysis. Firstly, no obvious heterogeneities were detected in overall analyses for two investigated polymorphisms, which indicated that eligible studies could be considered as homogeneous, and thus synthesize the results of these studies is statistically feasible. Secondly, the pathogenic mechanism of POI is highly complex, and hence it is unlikely that a single gene polymorphism can significantly contribute to its development. Therefore, to better illustrate potential correlations of certain gene polymorphisms with POI, we strongly recommend further studies to perform haplotype analyses and explore potential gene-gene interactions.

As with all meta-analysis, this study certainly has some limitations. First, our findings were based on unadjusted estimations due to lack of raw data, and failure to conduct further adjusted analyses for age, gender and co-morbidity conditions may impact the reliability of our findings [13, 14]. Second, association between *FSHR* polymorphisms and POI may also be influenced by gene-gene and gene-environmental interactions. However, the majority of studies did not consider these potential interactions, which impeded us to perform relevant analyses accordingly [15]. Third, only retrospective case-control studies were included in this meta-analysis, and thus direct causal relation between *FSHR* polymorphisms and POI could not be established. Taken these limitations into consideration, the results of the current study should be interpreted with caution.

## Conclusion

Overall, our meta-analysis suggests that the *FSHR* rs6166 polymorphism may serve as a potential genetic biomarker of POI in Asians, but not in other ethnicities. However, further well-designed studies with larger sample sizes are warranted to confirm our findings. Additionally, future investigations are needed to explore the potential roles of other *FSHR* polymorphisms in the development of POI.

## Data Availability

The current study was based on results of relevant published studies.

## Abbreviations

CI: Confidence interval
FSHR: Follicle-stimulating hormone receptor
OR: Odds ratio
POI: Premature ovarian insufficiency
